# Morphological and physiological responses of two willow species from different habitats to salt stress

**DOI:** 10.1038/s41598-020-75349-2

**Published:** 2020-10-26

**Authors:** Shuang Feng, Lulu Ren, Hongwei Sun, Kun Qiao, Shenkui Liu, Aimin Zhou

**Affiliations:** 1 Key Laboratory of Saline-Alkali Vegetation Ecology Restoration (Northeast Forestry University), Ministry of Education, Harbin, 150040 China; 2 College of Life Sciences, Northeast Forestry University, Harbin, 150040 China; 3 College of Horticulture and Landscape Architecture, Northeast Agricultural University, Harbin, 150030 China; 4 The State Key Laboratory of Subtropical Silviculture, Zhejiang Agriculture and Forestry University, Lin’An, Zhejiang, 311300 China

**Keywords:** Plant physiology, Plant stress responses

## Abstract

Plant salt tolerance is a complex mechanism, and different plant species have different strategies for surviving salt stress. In the present study, we analyzed and compared the morphological and physiological responses of two willow species (*Salix linearistipularis *and *Salix matsudana*) from different habitats to salt stress. *S. linearistipularis* exhibited higher seed germination rates and seedling root Na^+^ efflux than *S. matsudana* under salt stress. After salt treatment, *S. linearistipularis* leaves exhibited less Na^+^ accumulation, loss of water and chlorophyll, reduction in photosynthetic capacity, and damage to leaf cell structure than leaves of *S. matsudana*. Scanning electron microscopy combined with gas chromatography mass spectrometry showed that *S. linearistipularis* leaves had higher cuticular wax loads than *S. matsudana* leaves. Overall, our results showed that *S. linearistipularis* had higher salt tolerance than *S. matsudana*, which was associated with different morphological and physiological responses to salt stress. Furthermore, our study suggested that *S. linearistipularis* could be a promising tree species for saline-alkali land greening and improvement.

## Introduction

Willows (genus *Salix*) originate in China and comprise about 330–500 species mostly distributed in the temperate and arctic zones of the Northern Hemisphere^1,2^. Because of their rapid growth, high biomass yield, and ease of propagation, willows are important wood resources for bioenergy production and afforestation^3,4^. Furthermore, willow species are widely used in phytoremediation practices^5^. The comparison of environmental adaptability between willows distributed in different habitats could contribute to their production and application in phytoremediation under different environmental conditions.

The species *Salix linearistipularis *(Franch.) K.S. Hao and *Salix matsudana* Koidz. are willow species naturally distributed in northeast China. The Chinese willow (*S. matsudana*) is one of the most widely distributed and commonly cultivated willow species in China^6^. Studies have shown that *S. matsudana* plays important roles in heavy metal phytoextraction^7^. The species *S. linearistipularis* is a woody plant naturally distributed on the Songnen Plain on saline-alkali soil in the northeast of China^8^. The distribution patterns of *S. linearistipularis* and *S. matsudana* in different habitats suggest there are differences in their ability to survive salt stress. If *S. linearistipularis* had higher salt tolerance potential than *S. matsudana*, it could be used in saline-alkali soil land greening and improvement. However, whether these two species differ in salt tolerance remains unclear.

Plant salt tolerance is a complex process involving morphological, physiological, and biochemical changes^9^. In general, salt tolerant species are able to avoid or reduce the deleterious effects of absorbed salts through special functional mechanisms, such as (i) higher Na^+^ extrusion ability from roots, and (ii) more effective control of Na^+^ concentration and distribution in cells and tissues^10^. While in salt sensitive species, the effect of salt stress on transpiration, photosynthesis, and growth can be observed quickly, salt tolerant species exhibit such changes at high stress conditions or after prolonged exposure to salt stress^10^. For example, salt tolerant diploid wheat (*Triticum monococcum*) exhibited higher seed germination and higher leaf water and chlorophyll contents than those of salt sensitive tetraploid wheat (*T. durum*) under salt stress^11^. The roots of salt tolerant species *Populus euphratica* showed stronger Na^+^ efflux capacity under salt stress than the roots of salt sensitive *P. popularis*^12^. Salt treatment caused a change in cuticular wax load in salt tolerant plant *Grewia tenax*^13^. Cuticular wax load on the leaf surfaces protects the plant from a variety of environmental pressures^14^. Increased wax load, which reduces cuticular permeability, contributes to water conservation^14^. These studies suggested that morphological and physiological parameters such as seed germination, leaf photosynthetic capacity, epidermal wax load, and root Na^+^ efflux capacity can be used for evaluating plant salt tolerance.

In the present study, we compared seed germination, root Na^+^ and K^+^ efflux, and leaf morphological changes of two willow species (*S. linearistipularis* and *S. matsudana*) under salt stress treatments. Furthermore, salt damage to leaf cell structure and photosynthetic capacity between the two species was compared, and cuticular wax patterns, components, and loads of the leaves of the two willow species were observed and analyzed.

## Results

### Seed germination and seedling root Na^+^ and K^+^ efflux of two willow under salt stress

**Figure 1:**
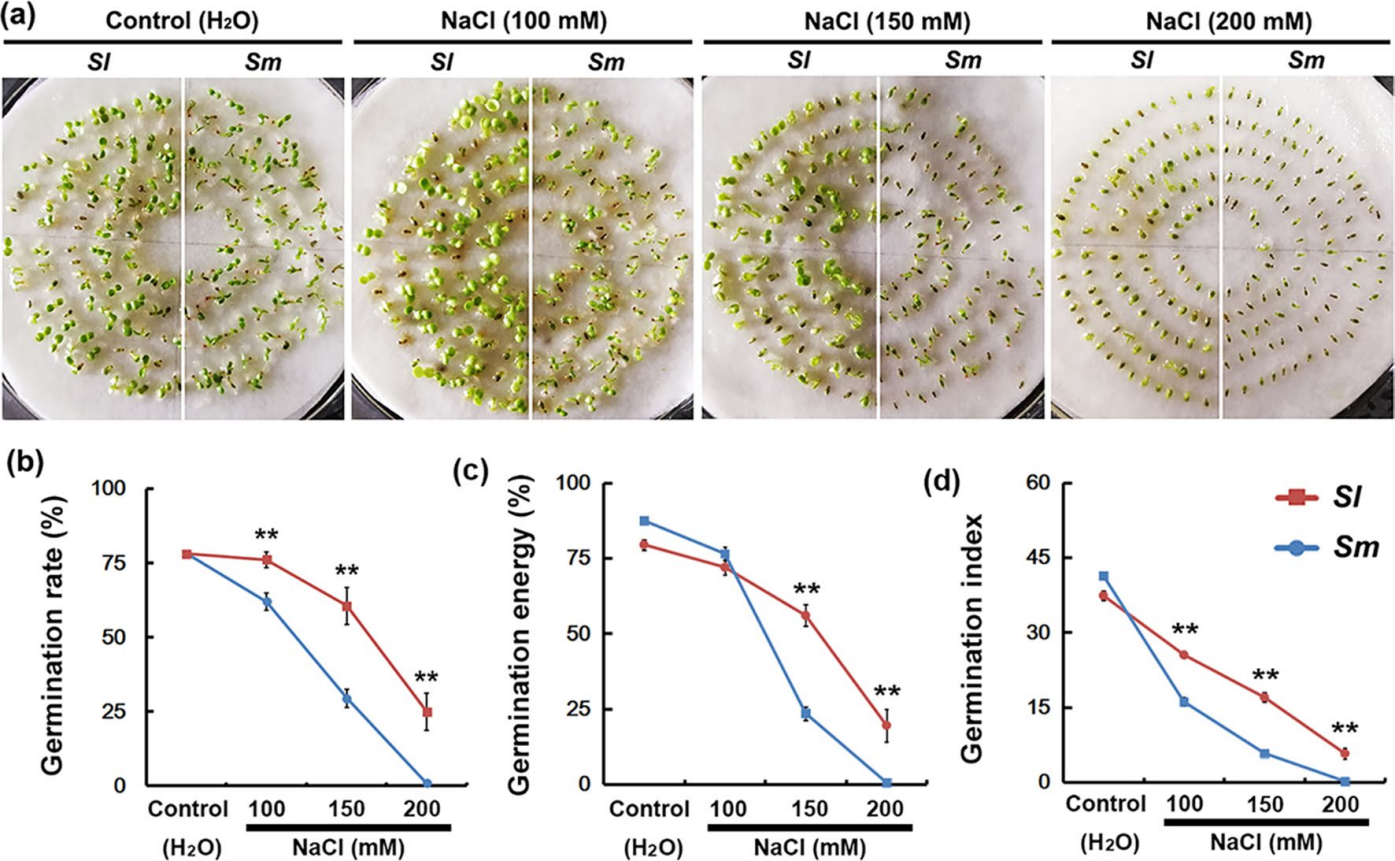
Comparison of seed germination rates between *S. linearistipularis* (*Sl*) and *S. matsudana* (*Sm*) under normal and salt stress conditions. (**a**) Seeds of *Sl* and *Sm* were germinated on filter paper containing aseptic water (control) or NaCl solution (100, 150, and 200 mM) for 4 days, and their germination rate (8 days) (**b**), germination energy (3 days) (**c**), and germination index (8 days) (**d**) were calculated. The asterisk indicates significant difference (**p < 0.01; Student’s *t* test). The error bar indicates *SE* (*n* = 6).

The germination rate, germination energy, and germination index of *S. linearistipularis* seeds was significantly higher than that of *S. matsudana* under treatments of 150 and 200 mM NaCl (Fig. 1a–d). Under 200 mM NaCl treatment, *S. linearistipularis* seeds still had a 25% germination rate, whereas *S. matsudana* seeds were completely unable to germinate. However, under untreated control conditions, their germination rate was similar (about 78%; Fig. 1a,b).

**Figure 2:**
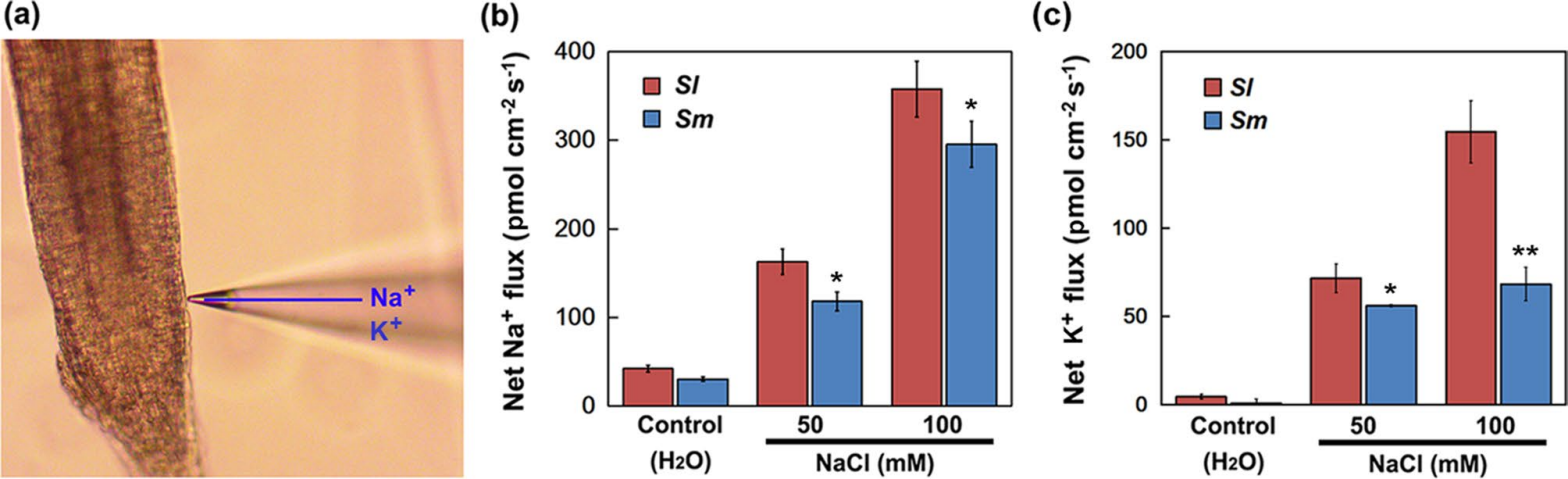
Comparison of root Na^+^ and K^+^ efflux rate between *S. linearistipularis* (*Sl*) and *S. matsudana* (*Sm*) seedling under normal and salt stress conditions. (**a**) Morphology and site of root monitored by NMT. Mean Na^+^ (**b**) and K^+^ (**c**) efflux rate from the roots of *Sl* and *Sm* seedlings (7-day-olds) after 12 h of incubation in aseptic water (control) or NaCl solutions (50 and 100 mM). The asterisk represents a significant difference (*p < 0.05; Student’s *t* test). The error bar indicates *SE* (*n* = 6).

Furthermore, Na^+^ and K^+^ flux in roots of *S. linearistipularis* and *S. matsudana* seedlings was compared by non-invasive micro-test technique (NMT) (Fig. 2a), and the results showed that NaCl treatment caused the increase of Na^+^ and K^+^ efflux rate in both seedling roots and that the Na^+^ and K^+^ efflux rate of *S. linearistipularis* roots was significantly higher than that of *S. matsudana* under salt stress. However, there was no significant difference between the Na^+^ and K^+^ efflux rates of the two species under control conditions in which both species exhibited weak Na^+^ and K^+^ efflux (Fig. 2b,c). These results suggested that the *S. linearistipularis* has higher seed germination rate and seedling root Na^+^ and K^+^ efflux capacity under salt stress than those of *S. matsudana*.

### Na^+^ and K^+^ content and water loss of two willow species leaves under salt stress

**Figure 3:**
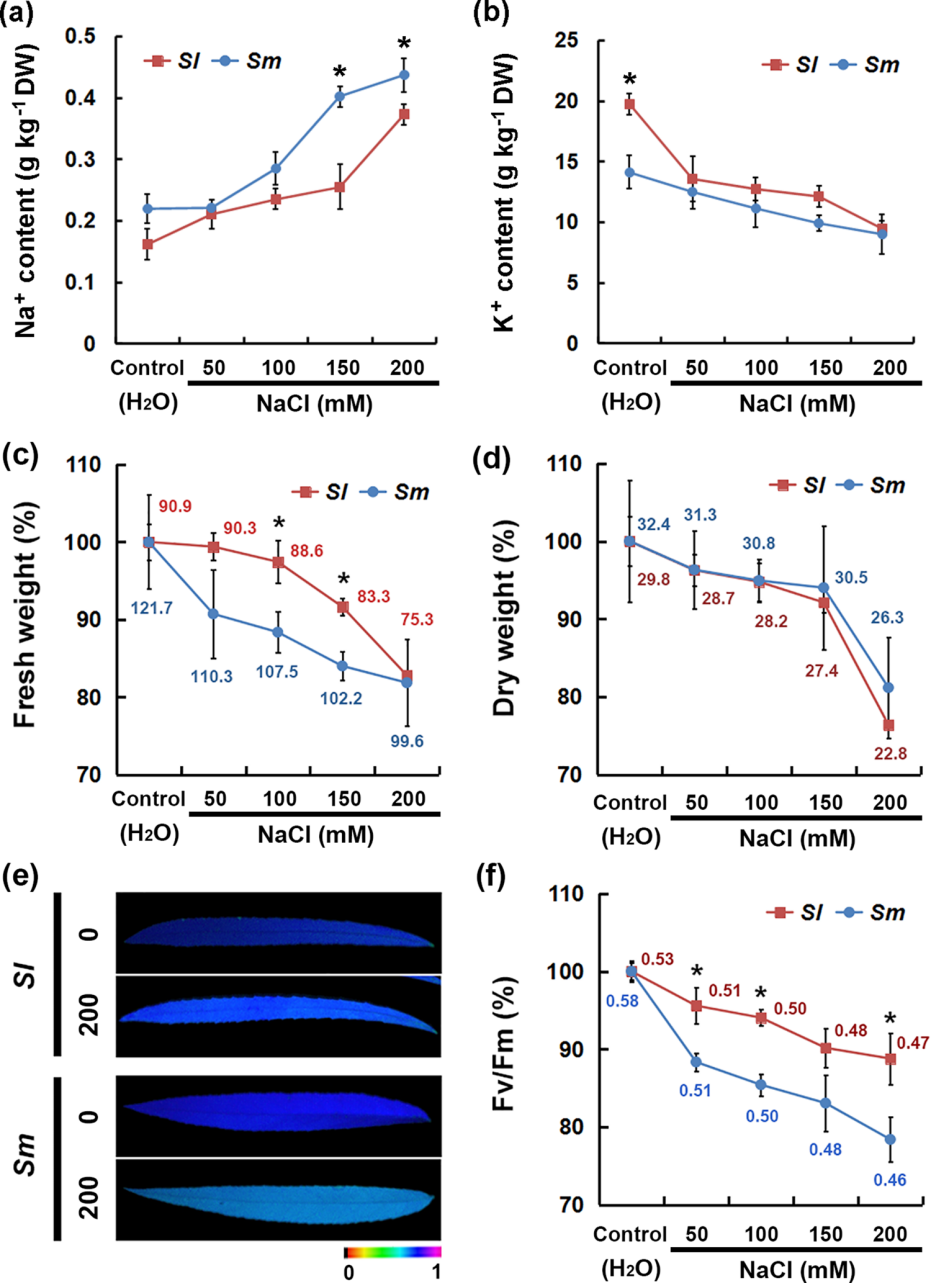
Comparison of seedling phenotypes between *S. linearistipularis* (*Sl*) and *S. matsudana* (*Sm*) under normal and salt stress conditions. Na^+^ (**a**) and K^+^ (**b**) content, fresh (**c**) and dry (**d**) weight, and maximal photochemical efficiency (Fv/Fm) (**e**,**f**) from the leaves of *Sl* and *Sm* seedlings (2-month-olds) treated with aseptic water (control) or NaCl solutions (50, 100, 150, and 200 mM) for 3 days. In (**c**,**d**,**f**), red and blue numbers indicate the actual measured value of *Sl* and *Sm* leaves, respectively. The asterisk represents a significant difference (*p < 0.05; Student’s *t* test). The error bar indicates *SE* (*n* = 6).

The effects of salt stress on the seedlings growth of two willow species were compared. After NaCl treatments (150 and 200 mM), the Na^+^ content in *S. linearistipularis* leaves was lower than that in *S. matsudana* leaves, while the K^+^ content was slightly higher than that in *S. matsudana* leaves (Fig. 3a,b). Furthermore, *S. linearistipularis* leaves exhibited less reduction in fresh weight and maximal photochemical efficiency (Fv/Fm) than *S. matsudana* leaves, but they do not showed difference in dry weight (Fig. 3c–f). These results suggested that the *S. linearistipularis* leaves has less Na^+^ accumulation and water loss under salt stress than those of *S. matsudana*.

### Photosynthetic parameters and leaf ultrastructure of two willow species leaves under salt stress

**Figure 4:**
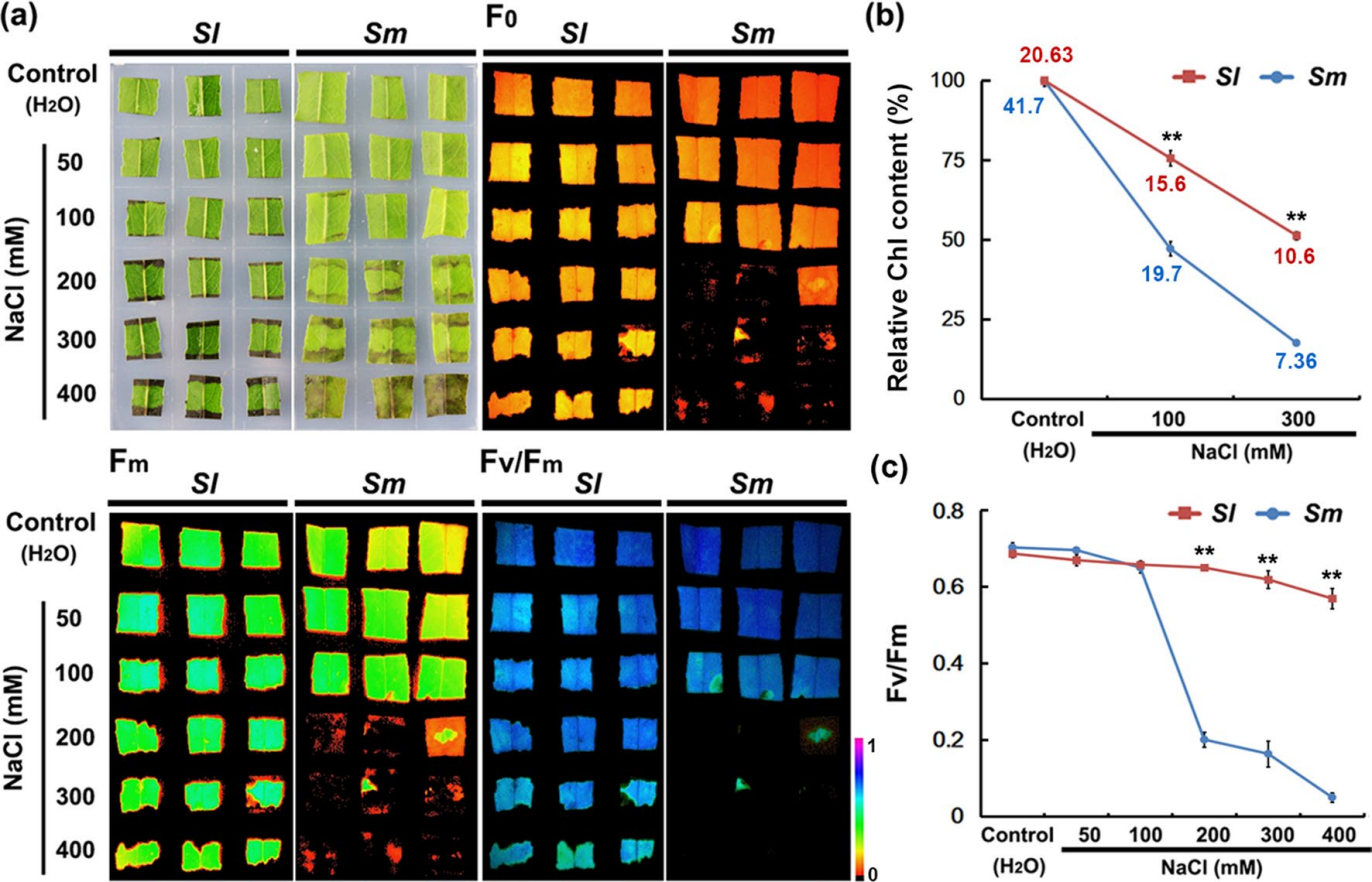
Comparison of leaf morphology and photosynthetic parameters between *S. linearistipularis* (*Sl*) and *S. matsudana* (*Sm*) under normal and salt stress conditions. The leaf discs (1–1.5 cm^2^) were immersed in aseptic water (control) or NaCl solution at different concentrations (50 to 400 mM) for 48 h. (**a**) The morphology of leaf discs, and their minimum Chl fluorescence (F_0_), maximal Chl fluorescence (Fm), and maximal photochemical efficiency (Fv/Fm) images. The colored bar at the bottom indicates the panel range from 0 (black) to 1.0 (purple). (**b**) Relative chlorophyll (Chl) content. **(c)** Fv/Fm values. In (**b**), red and blue numbers indicate the actual measured chlorophyll content of *Sl* and *Sm* leaves, respectively. The asterisk represents a significant difference (**p < 0.01; Student’s *t* test). Error bars represent *SE* (*n* = 9).

In order to investigate the difference in salt tolerance between the two willow species, their leaf morphology under salt stress was compared. After NaCl treatments (200, 300, and 400 mM), the area of *S. matsudana* leaves losing green color was generally larger than that of *S. linearistipularis* leaves (Fig. 4a). The analysis of relative chlorophyll (Chl) content showed that the decrease of Chl in *S. matsudana* leaves was significantly higher than that in *S. linearistipularis* leaves after NaCl treatment (Fig. 4b). Photosynthesis parameter measurements showed that the Fv/Fm value of *S. matsudana* leaves was significantly lower than that of *S. linearistipularis* leaves after NaCl treatments with more than 200 mM (Fig. 4a,c). These results suggested that the damage of salt to leaf photosynthetic capacity was significantly higher in *S. matsudana* than that in *S. linearistipularis*.

**Figure 5:**
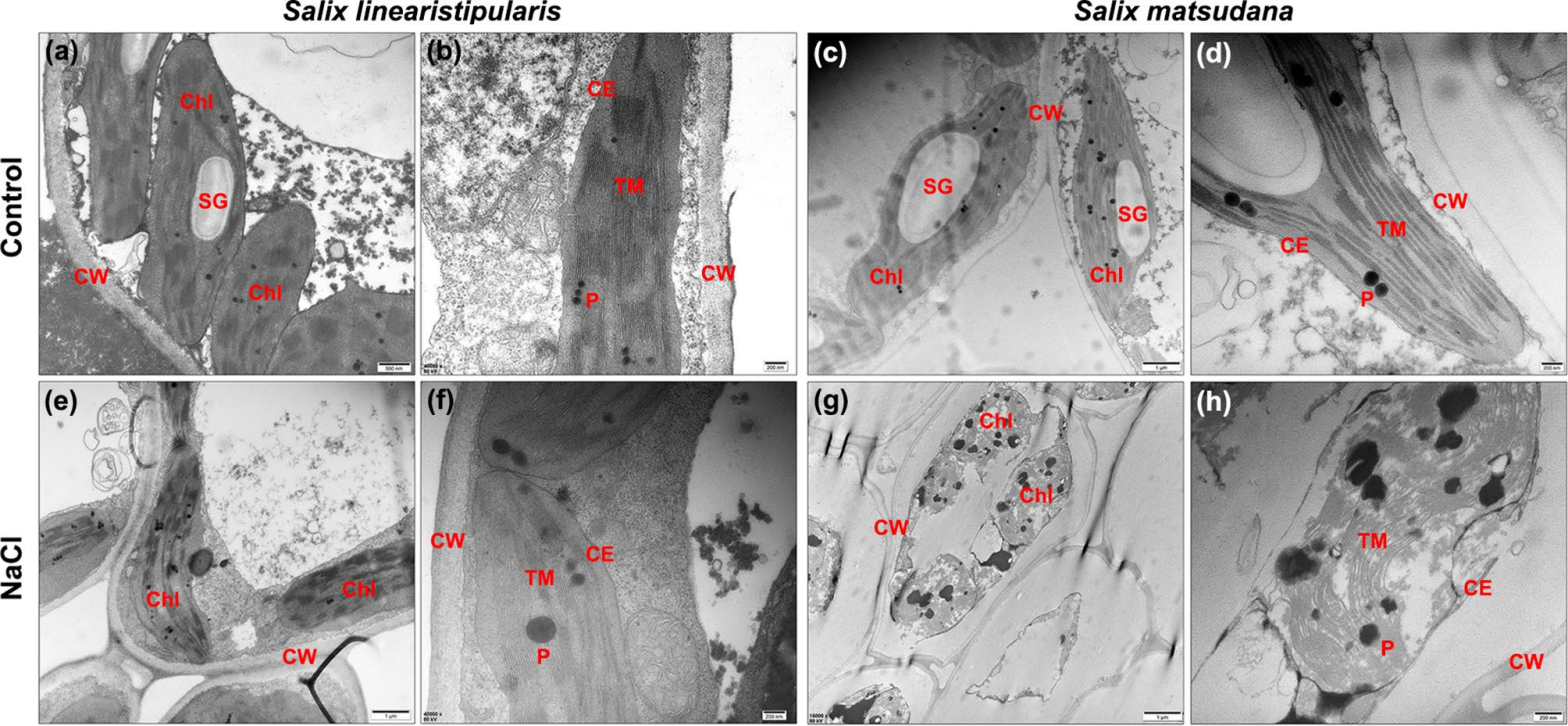
Comparison of TEM images of leaf cell ultrastructure between *S. linearistipularis* and *S. matsudana* under (**a**–**d**) control and (**e**–**h**) salt stress conditions (300 mM NaCl for 48 h) *CW* cell wall, *Chl* chloroplast, *CE* chloroplast envelope, *TM* thylakoid membrane, *P* plastoglobulus, *SG* starch granule. Error bar = 1 μm (**c**,**e**,**g**), 500 nm (**a**), and 200 nm (**b**,**d**,**f**,**g**).

Photosynthetic capacity is directly related to chloroplast structure and function. Under salt stress, the ultrastructure of cells in *S. linearistipularis* and *S. matsudana* leaves, especially particularly chloroplasts, was compared by TEM. TEM images showed similar healthy cellular structures in *S. linearistipularis* and *S. matsudana* leaves under control conditions (Fig. 5a–d). After NaCl (300 mM) treatment, *S. linearistipularis* leaf cell structure remained unchanged, whereas *S. matsudana* leaf cell structure was almost completely damaged (Fig. 5e–h). In *S. matsudana* leaf cells, we observed severe cytoplasmic wall separation, damaged chloroplast envelopes (CE) and thylakoid membranes (TM), and diffuse plastoglobuli (Fig. 5g,h). These results suggested that the damage of salt to leaf cell ultrastructure in *S. matsudana* was more serious than that of in *S. linearistipularis*.

### Leaf crystal patterns and thickness of cuticular wax in two willow species leaves

**Figure 6:**
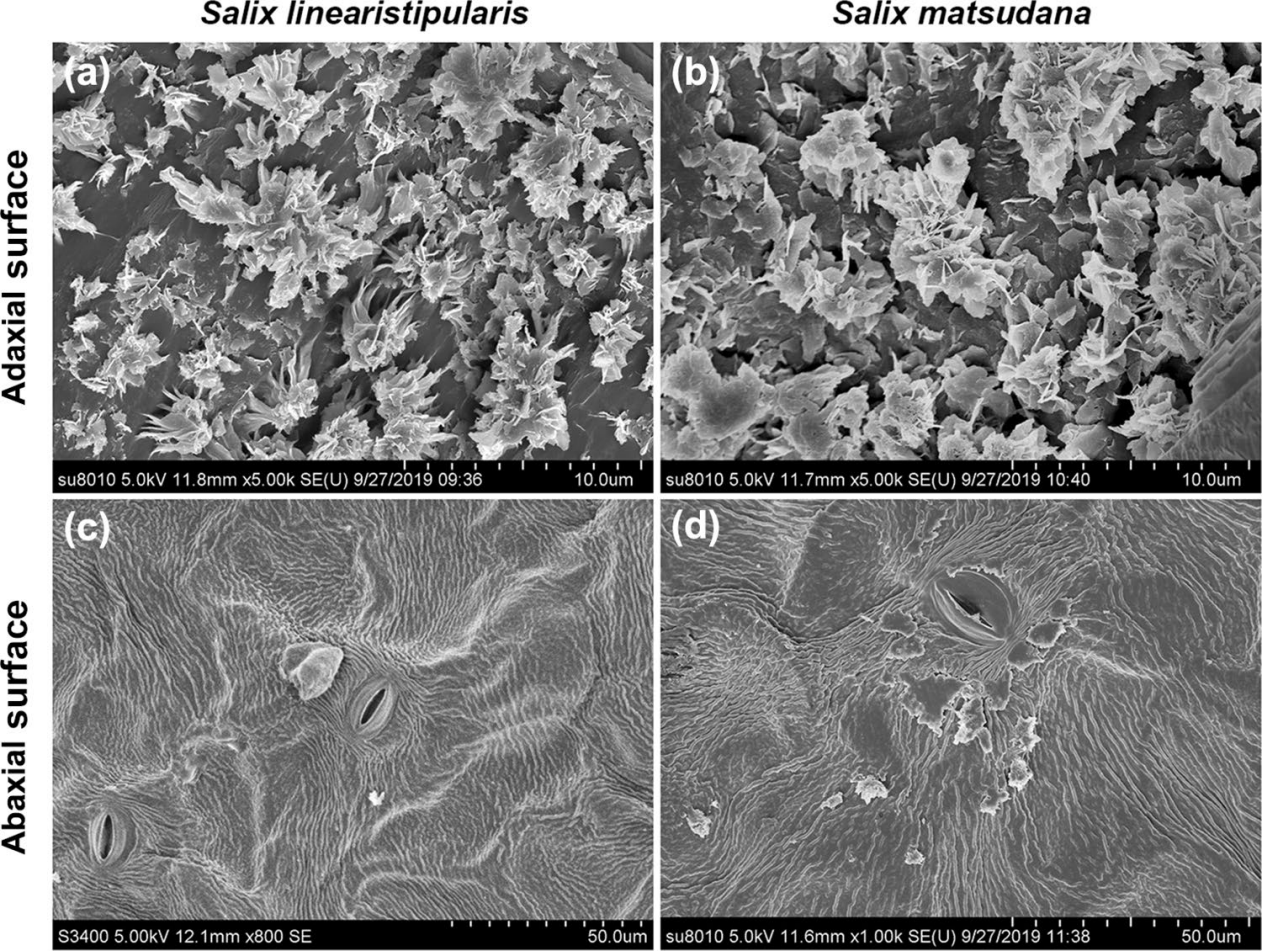
Comparison of cuticular wax crystal patterns on the leaf surfaces between *S. linearistipularis* and *S. matsudana*. SEM images of (**a**,**b**) the adaxial and (**c**,**d**) abaxial surfaces of *Sl* and *Sm* leaves. Error bar = 10 μm (**a**,**b**), 50 μm (**c**,**d**).

As salt damage to leaf cell structure may be related to the permeability of leaf surface, we observed and compared the adaxial (upper) and abaxial (lower) surfaces of the two willow species leaves. SEM images showed that the adaxial surface of both *S. linearistipularis* and *S. matsudana* leaves has a waxy structure, and the waxy structures were highly similar in shape and density (Fig. 6a,b). However, the abaxial surface of both leaves was smooth (Fig. 6c,d).

**Figure 7:**
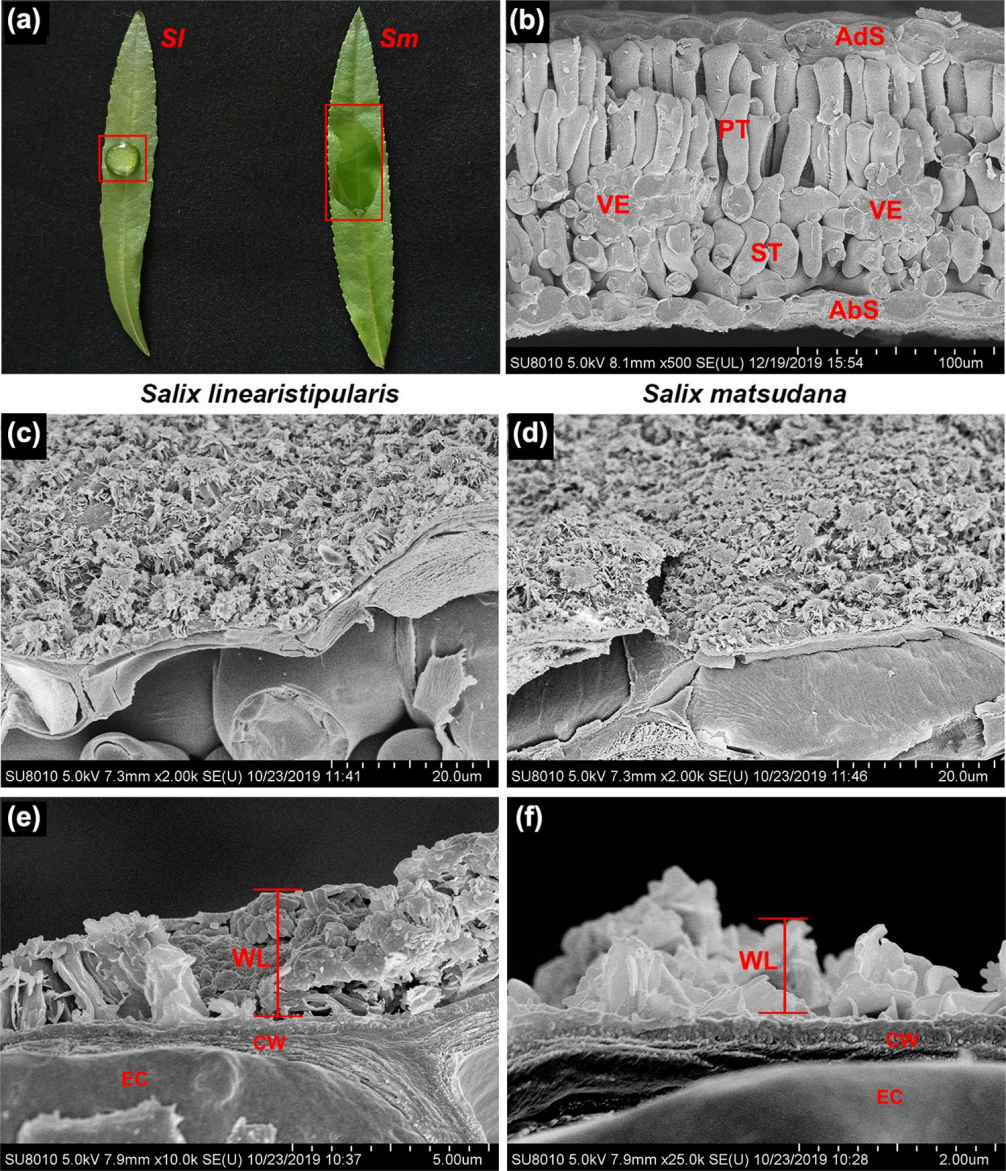
Comparison of cuticular wax layer thickness on the leaf surfaces of *S. linearistipularis* (*Sl*) and *S. matsudana* (*Sm*). (**a**) Water adhesive phenotype of *Sl* and *Sm* leaves. (**b**) Cryo-SEM image of leaf cross sections. (**c**–**f**) Cryo-SEM images of the cuticular wax layers of *Sl* (**c**,**e**) and *Sm* (**d**,**f**) on leaf adaxial surface. Red bars indicate the thickness of the cuticular wax layer. *AdS* adaxial surface, *AbS* abaxial surface, *PT* palisade tissue, *ST* spongy tissue, *VE* vein, *WL* wax layer, *EC* epidermal cell, *CW* cell wall. Error bar = 100 μm (**a**), 20 μm (**c**,**d**), 5 μm (**e**), and 2 μm (**f**).

Water adhesion analysis showed that on the adaxial surface of *S. linearistipularis* leaves, less water droplets were formed by the same volume of water than *S. matsudana* leaves (Fig. 7a), and therefore we speculated that the thickness of the wax layer on leaf surface differed between the two species. Furthermore, cross sections of leaves were observed using cryo-SEM, and the results showed that the willows had typical bifacial leaves (Fig. 7b) with epidermal waxes accumulated on the adaxial surface in the leaves of both species (Fig. 7c,d). A large number of our observations found that the thickness of the wax layer on the adaxial surface of *S. linearistipularis* leaves was generally higher than that of *S. matsudana* leaves (Fig. 7e,f). These results suggested that the crystal pattern of the cuticular wax of the two willow leaves was highly similar, but the thickness of the wax differed between the two investigated species.

### Components and loads of cuticular wax in two willow species leaves

**Figure 8:**
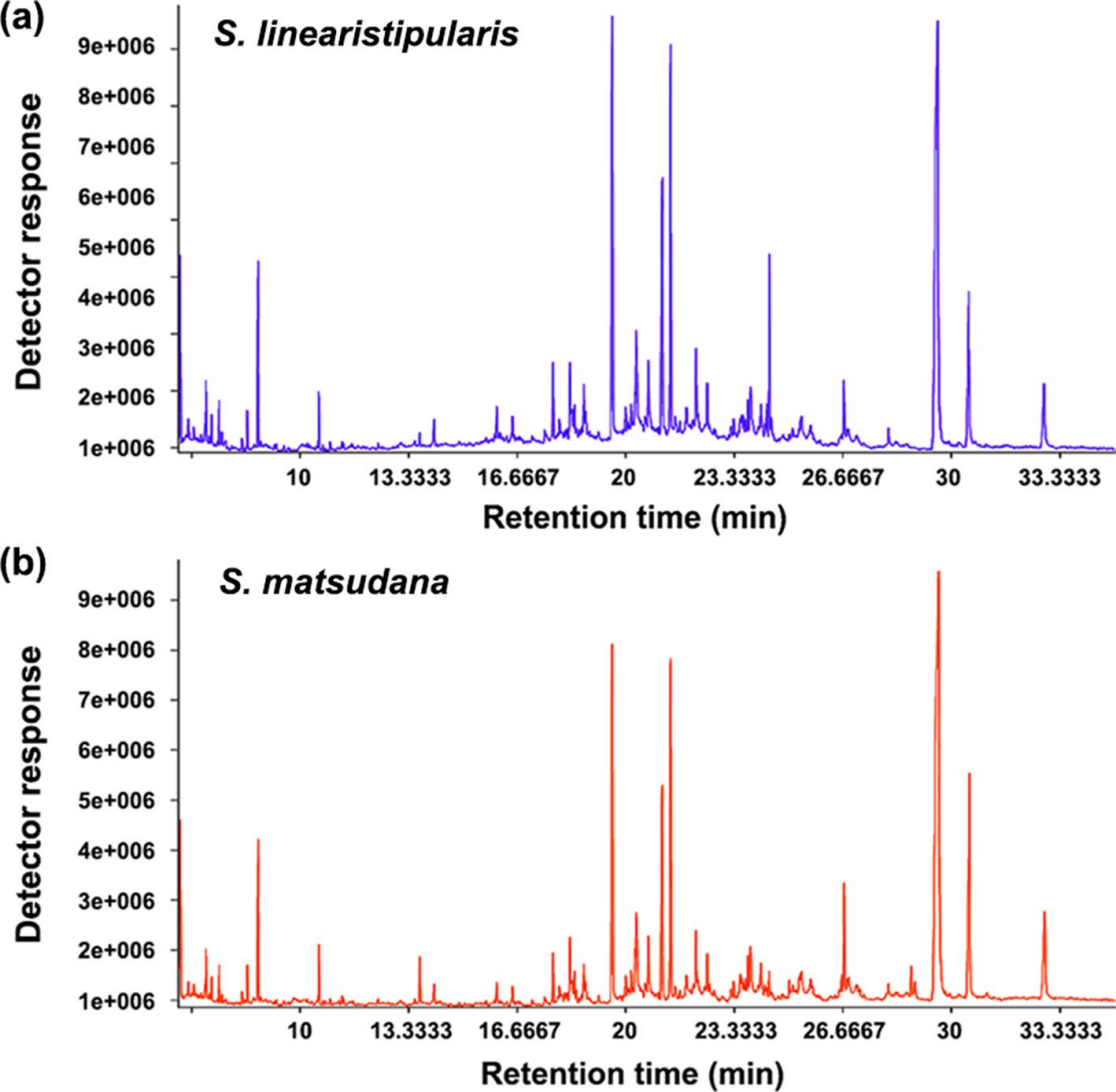
Total ion chromatograms (TIC) of cuticular wax components of (**a**) *S. linearistipularis* and (**b**) *S. matsudana* (*Sm*) leaves.

**Figure 9:**
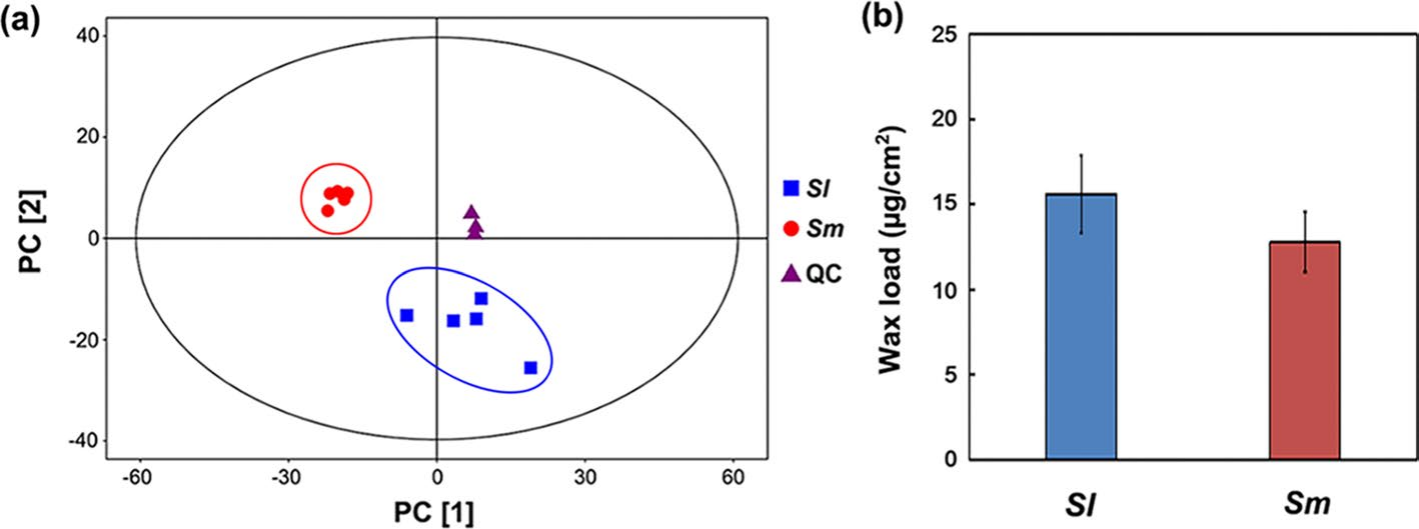
Comparison of cuticular wax loads on the leaf surfaces of *S. linearistipularis* (*Sl*) and *S. matsudana* (*Sm*). (**a**) Results of the principal component analysis of the wax components of *Sl* and *Sm* leaves. *QC* quality control. The 95% confidence interval region according to Hotelling’s T2 statistic. (**b**) Cuticular wax loads of *Sl* and *Sm* leaves. Error bars represent *SE* (*n* = 5).

Cuticular wax components from the leaves of the two willow species were extracted and analyzed. GC–MS revealed that the cuticular wax components of the two leaves were constituted of many chemical compounds, including fatty acids, alcohols, and alkanes (Fig. 8). These major components were identified by corresponding retention times and similarity matching scores (more than 600; Table 1). The results of the principal component analysis showed that the cuticular wax loads of the two leaves were well distinguished in content (Fig. 9a). Statistical analysis showed that the cuticular wax load of *S. linearistipularis* leaves was higher than that of *S. matsudana* leaves (Fig. 9b).

**Table 1:** Cuticular wax components identified from *S. linearistipularis* and *S. matsudana* leaves.

Class	Peak^a^	Retention time (min)	Similarity^b^	Formula
Fatty acids	Palmitic acid	19.5869	933	C_16_H_32_O_2_
	Linolenic acid	21.1306	841	C_18_H_30_O_2_
	Linoleic acid	21.0693	600	C_18_H_32_O_2_
	Stearic acid	21.3773	933	C_18_H_36_O_2_
	Arachidic acid	22.9964	830	C_20_H_40_O_2_
	Lignoceric acid	25.9542	829	C_24_H_48_O_2_
	Cerotinic acid	27.2949	862	C_26_H_52_O_2_
Alcohols	Octadecanol	20.5803	684	C_18_H_38_O
Alkanes	Tetracosane	22.7024	866	C_24_H_50_

^a^Peak indicates the name of the substance according to the Fiehn database.

^b^Similarity indicates the matching (0 to 1000) of the substance to the mass spectrometry peak.

## Discussion

Wild species inhabiting natural saline habitats possess genetic variations which are the basis of the evolution of salt tolerant populations^15–17^. *S. linearistipularis* is a woody plant naturally distributed in the saline-alkaline lands with high salinity in the Songnen plain of northeast China, showing its strong salt adaptability^8^. In the present study, we investigated the morphological and physiological characteristics associated with salt tolerance in *S. linearistipularis* and compared them to those of *S. matsudana*. Under salt stress, *S. linearistipularis* exhibited higher seed germination rate and higher seedling root Na^+^ efflux capacity than that of *S. matsudana* (Figs. 1, 2). Similarly, the Na^+^ efflux in roots of salt tolerant *P. euphratica* was significantly higher than that of salt sensitive *P. popularis* under salt stress^12^. Furthermore, *S. linearistipularis* leaves had less Na^+^ accumulation under salt stress than *S. matsudana* leaves (Fig. 3a). Higher Na^+^ efflux in roots may reduce Na^+^ accumulation and its toxic effects under salt stress. Limiting the entry of salt (mainly Na^+^) by the roots and maintaining lower Na^+^ accumulation in tissues or cells is one of the main salt tolerance strategies evolved by plants^18,19^. These results suggested that the more active root Na^+^ efflux capacity and less leaf Na^+^ accumulation of *S. linearistipularis* seedlings under salt stress contributed to its salt tolerance.

Salt also causes injuries of the young photosynthetic leaves and accelerates their senescence^19^. In the present study, salt treatment caused a decrease in Chl content and photosynthetic capacity (indicated by Fv/Fm parameters) in the leaves of both willow species; however, *S. linearistipularis* leaves maintained higher Chl content and Fv/Fm value compared to those of *S. matsudana* leaves after salt treatment (Fig. 4). Furthermore, TEM analysis showed that the damage to *S. linearistipularis* leaf cell structures caused by the salt treatment, particularly, the damage to the photosynthetic membrane structures in chloroplasts, was significantly lower than that to *S. matsudana* leaves (Fig. 5e–h). These results suggested that less damage to leaf cell structure, chlorophyll loss, and reduction in photosynthetic capacity of *S. linearistipularis* leaves under salt stress contributed to its salt tolerance. However, the response of isolated leaves to salt may be different from that of living plants. Therefore, the morphological and physiological responses of leaves from two living willow plants under salt stress need further investigation and comparison.

Salt stress causes osmotic stress, resulting in the loss of water from plant leaves^20^. Plant cuticular waxes play a crucial role in limiting non-stomatal water loss in leaves^21^. In the present study, SEM analysis showed that the cuticular waxes were present on the adaxial surfaces of the leaves of both willow species, and the waxes had highly similar crystal patterns (Fig. 6a,b). However, cryo-SEM analysis showed that the thickness of the cuticular waxes in *S. linearistipularis* leaves was generally higher than that in *S. matsudana* leaves (Fig. 7e,f), which was also confirmed by GC–MS analysis (Fig. 9). Cuticular wax load was found to be negatively correlated with leaf water loss rate^22,23^. Fresh weight measurements showed that the water loss ratio of *S. linearistipularis* leaves under salt stress was lower than that of *S. matsudana* leaves (Fig. 3c). These results suggested that higher cuticular wax loads of *S. linearistipularis* leaves than those of *S. matsudana* leaves contributed to its higher salt tolerance. Different environmental conditions can affect the distribution and chemical composition of cuticular waxes in plants^24–26^. Thus, we speculated that the saline-alkali conditions affected the cuticular wax loads in *S. linearistipularis* leaves. Studies have shown that several species of the genus *Salix* differ in cuticular wax loads, but have highly similar wax composition^27,28^.

Overall, our study showed that compared to *S. matsudana*, *S. linearistipularis* has higher salt tolerance, which is associated with higher root Na^+^ efflux, less leaf Na^+^ accumulation, better maintenance of leaf cell structure and photosynthetic capacity, and higher cuticular wax load under salt stress conditions. Our results suggest that *S. linearistipularis* could be a promising tree species for saline-alkali land greening, improvement, and phytoremediation practices.

## Materials and methods

### Plant material

In May 2019, seeds of *S. linearistipularis* and *S. matsudana* were collected from the saline-alkali land of the Songnen Plain (Anda City, Heilongjiang Province, China; 46° 27′ N, 125° 22′ E) and the experimental base of Northeast Forestry University, respectively.

### Seed germination test

Fifty seeds from two willows were sown on plates with filter paper containing aseptic water (control) or NaCl solutions of different concentrations (100, 150 and 200 mM). These seeds were cultured for 8 days at 22 °C before measuring seed germination rate, germination energy, and germination index. The experiment was replicated four times.

### Measurement of Na^+^ and K^+^ efflux

Seedlings grown in aseptic water were used for the measurement of Na^+^ and K^+^ efflux. Hydroponic seedlings (7-day-olds) were exposed to aseptic water (control) or NaCl solution (50 and 100 mM) for 12 h, and root segments were immobilized in the measuring solution (0.1 mM KCl, 0.1 mM CaCl_2_, 0.1 mM MgCl_2_, 0.5 mM NaCl, and 0.3 mM MES, pH 5.8) in order to measure the Na^+^ flux. Net fluxes of Na^+^ and K^+^ were measured using the non-invasive micro-test technique (NMT100 Series, YoungerUSA LLC, Amherst, MA, USA) as described in^12,29^.

### Measurement of Na^+^ and K^+^ contents

Two-month-old seedlings grown in soil (1 L) were irrigated with different concentrations (0, 50, 100, 150, and 200 mM NaCl) of salt solution (500 mL) for 3 days. After salinity treatment, the leaf samples of seedlings were collected, weighed and dried. The dried samples were weighed and then digested in 8 mL HNO_3_ and 3 mL H_2_O_2_ for 50 min at 180 °C using a microwave digestion instrument (Milestone, Italy). The Na^+^ and K^+^ contents in the leaves were measured by inductively coupled plasma optical emission spectrometry (ICP-OES, Perkin Elmer, USA).

### Measurement of chlorophyll content and chlorophyll fluorescence parameters

The leaves were shaped into leaf discs (1 cm^2^) which were immediately immersed in aseptic water (control) or NaCl solutions of different concentrations (50, 100, 200, 300 and 400 mM) for 48 h. Chlorophyll (Chl) was extracted from the leaf samples with 80% ice-cold acetone. The absorbances of Chl a (646 nm) and Chl b (663 nm) were determined using a UV/Vis spectrophotometer. The total Chl content was calculated as the sum of Chl a and Chl b. The maximal photochemical efficiency (Fv/Fm), minimal fluorescence yield (F_0_), and maximal fluorescence yield (Fm) were measured using an Imaging-PAM Chlorophyll Fluorometer (Walz, Germany) as described in^30^.

### Transmission electron microscopy (TEM)

The untreated leaf discs and leaf discs treated with NaCl (300 and 400 mM) were incubated in the fixation solution (2.5% [v/v] glutaraldehyde in 0.1 M phosphate buffer, pH 7.4) under vacuum conditions for 4 h. The leaf discs were then dehydrated in ethanol and embedded in LR White resin (Sigma-Aldrich). Polymerization and ultrathin section (60–80 nm) were obtained as previously described^31^. The sections were used for electron microscopy with H-7500 transmission electron microscope (Hitachi, Tokyo, Japan) which was set at 80 kV.

### Scanning electron microscopy (SEM and cryo-SEM)

SEM and cryo-SEM analyses were performed as previously described^22^. For SEM analysis, leaf samples were collected, fixed with glutaraldehyde buffer, and gradually dehydrated using alcohol. The leaf samples were then dried to the critical point using liquid CO_2_ and sputter coated with an electrically conductive gold layer before being imaged by SEM (Hitachi SU-8010, Tokyo, Japan) at 5 kV.

For cryo-SEM analysis, leaf samples were sprinkled onto a perforated aluminum stub and plunged into liquid nitrogen slush (− 210 °C). The frozen samples were transferred to a cryo system (PP3010T; Quorum Technologies, Lewes, UK), sputter coated with platinum, transferred to the SEM cold stage, and examined at − 140 °C at a beam voltage of 5 kV and probe current of 10 mA.

### Wax extraction

Leaf samples were placed into a 50 mL tube and 30 mL pre-cold extraction mixture (chloroform) was added. The samples were vortexed for 30 s and ultrasonicated in water for 30 min at 60 ± 5 °C. The samples were then taken out of the tubes and 20 μL of internal standard (adonitol, 0.5 mg mL^−1^ stock) was added to each tube. The samples were nitrogen blow-dried and reconstituted in 5 mL of chloroform by sonication on ice for 5 min. After centrifugation (4 °C, 10 min, 5000 rpm), 500 μL of the supernatant was transferred to a new tube. In order to prepare the Quality control (QC) sample, 150 μL of each sample was taken and these samples were combined.

After evaporation in a vacuum concentrator, 50 μL of methoxyamination hydrochloride (20 mg mL^−1^ in pyridine) was added and incubated at 80 °C for 30 min, and derivatization was achieved by dissolving the samples in 70 μL of BSTFA reagent (1% TMCS, v/v) at 70 °C for 1.5 h. The samples were gradually cooled to room temperature, and 5 μL of FAMEs (in chloroform) was added to the QC sample. All samples were analyzed by gas chromatograph coupled with a time-of-flight mass spectrometer (GC-TOF-MS)^32^.

### GC-TOF-MS analysis

GC-TOF-MS analysis was performed using an Agilent 7890 gas chromatograph coupled with a time-of-flight mass spectrometer as previously described^32^. The system utilized a DB-5MS capillary column. A volume of 1 μL of sample aliquot was injected in splitless mode. Helium was used as the carrier gas, the front inlet purge flow was 3 mL min^−1^, and the gas flow rate through the column was 1 mL min^−1^. The initial temperature was kept at 50 °C for 1 min, then raised to 310 °C at a rate of 10 °C min^−1^ and kept at 310 °C for 8 min. The injection, transfer line, and ion source temperatures were 280 and 250 °C, respectively. The energy was − 70 eV in electron impact mode. The mass spectrometry data were acquired in full-scan mode with the m/z range of 50–500 at a rate of 12.5 spectra per second after a solvent delay of 6.25 min.

### Data preprocessing

Raw data analysis, including peak extraction, baseline adjustment, deconvolution, alignment, and integration, was performed with Chroma TOF software (V 4.3x, LECO)^33^, and LECO-Fiehn Rtx5 database was used for metabolite identification by matching the mass spectrum and retention index. Finally, the peaks detected in less than half of QC samples or RSD > 30% in the QC samples were removed^34^.

### Statistical analyses

The data were analyzed using one-way analysis of variance by SPSS software, and statistically significant differences were calculated based on Student’s *t*-test, with *p* < 0.05 (*) and *p* < 0.01 (**) as the thresholds for significance^22^.
